# Clinical efficacy of mineralized collagen (MC) versus anorganic bovine bone (Bio-Oss) for immediate implant placement in esthetic area: a single-center retrospective study

**DOI:** 10.1186/s12903-021-01752-4

**Published:** 2021-08-10

**Authors:** Yan Dai, Jin Xu, Xiao-Hui Han, Fu-Zhai Cui, Dong-Sheng Zhang, Sheng-Yun Huang

**Affiliations:** 1 Department of Oral and Maxillofacial Surgery, Zibo Central Hospital, Zibo, 255036 China; 2 Department of Basic Medicine, Kangda College of Nanjing Medical University, Lianyungang, 222000 China; 3 Department of Prosthodontics, Shandong Provincial Hospital Affiliated to Shandong First Medical University, Jinan, 250021 Shandong China; 4 State Key Laboratory of New Ceramics and Fine Processing, School of Materials Science and Engineering, Tsinghua University, Beijing, 100084 China; 5 Department of Oral and Maxillofacial Surgery, Shandong Provincial Hospital Affiliated to Shandong First Medical University, Jinan, 250021 Shandong China

**Keywords:** Mineralized collagen, Immediate implant implantation, Osseointegration

## Abstract

**Background:**

The purpose of this retrospective study was to evaluate the clinical efficacy of mineralized collagen (MC) versus anorganic bovine bone (Bio-Oss) for immediate implant placement in esthetic area.

**Methods:**

Medical records of Department of Oral and Maxillofacial Surgery of Shandong Provincial Hospital were screened for patients who had been treated with immediate implant implantation in the esthetic area using either MC (Allgens®, Beijing Allgens Medical Science and Technology Co., Ltd., China) or Bio-Oss (Bio-Oss®, Geistlich Biomaterials, Wolhusen, Switzerland), between January 2018 and December 2019. All patients fulfilling the in-/exclusion criteria and following followed for a minimum period of 1 year after surgery were enrolled into the presented study. Implant survival rate, radiographic, esthetic and patient satisfactory evaluations were performed.

**Results:**

Altogether, 70 patients were included in the study; a total of 80 implants were inserted. All implants had good initial stability. The survival rate of implants was 100% at 1-year follow-up. The differences in horizontal and vertical bone loss between the MC group (0.72 ± 0.26 mm, 1.62 ± 0.84 mm) and the Bio-Oss group (0.70 ± 0.52 mm, 1.57 ± 0.88 mm) were no significant difference statistically no significant 6 months after permanent restoration. Similar results occurred at 12 months after permanent restoration functional loaded. Clinical acceptability defined by pink esthetic score (PES) ≥ 6 (6.07 ± 1.62 vs. 6.13 ± 1.41) was not significantly different between groups. Patient satisfaction estimated by visual analog scale (VAS) was similar (8.56 ± 1.12 vs. 8.27 ± 1.44), and the difference was no significant difference between the two groups.

**Conclusions:**

The biomimetic MC showed a similar behaviour as Bio-Oss not only in its dimensional tissues changes but also in clinical acceptability and patient satisfaction. Within the limitations of this study, these cases show that MC could be considered as an alternative bone graft in IIP

## Background

To simplify the surgical procedures and treatment time, clinicians and researchers have developed a new implant placement protocol, defined as "immediate implant implantation (IIP)” [1]. As the name implies, IIP means that placing a dental implant immediately into fresh extraction sockets. Since the first report of IIP published in 1976, the interest for this technique has gradually increased [2]. Studies suggest that IIP may provide some advantages, such as preventing alveolar ridge absorption, maintaining the width and height of alveolar crest, getting satisfactory esthetic results. Finally, the implant can be seated in the ideal anatomical position according with the biomechanics [3–14]. Furthermore, several clinical Studies have shown that that similar implant survival rates are independent of the timing of implant placement [15–17]. Based on the systematic review, patients prefer short treatment protocols to conventional implant placement [18]. Consequently, IIP is recognized by clinicians nowadays. However, the size of the implant do not completely fit with the socket, and particularly, the residual gap between the implants and the labial alveolar cannot fulfill a tight closure affecting the success of IIP. The gap provides sufficient space to fill the bone defect between the labial bone wall and the exposed implant surface with suitable bone filler, and it also provides space for formatting of a blood clot, which can subsequently restructure into a provisional matrix of connective tissue that support the newly-formed woven bone. Pro.Tarnow recommended to place a bone graft into the residual labial gap around a postextraction socket anterior implant. It’s helpful for limiting the amount of facial-palatal contour change in IIP [19]. Various bone graft materials have been employed around IIP for bone augmentation aiming to promote the tissue which contained osteogenic cells to fill out the defect area [20]. Bone grafting materials, categorized into autogenous, allografts, xenografts, and alloplasts, have been applied to fill the osseous gap around the implant [21]. Till date, no literature has proved that one material is superior to another [22–25].

MC designed by Cui et al. is a novel form of artificial bone graft, which consists of orderly arranged type I collagen and nano-hydroxyapatite (HA) [26]. It contains approximately 45% mineral by weight. This material possesses excellent biological histocompatibility and osseointegration with a capability of biomimetic composition and microstructure similar to the natural bone tissue. As demonstrated in previous studies, MC had been successfully applied for bone defect repair in a wide range. Peng et al. observed the effect of MC for the treatment of senile proximal humeral osteoporotic fractures, proving that it can accelerate healing of senile proximal humeral fracture, improve the therapeutic effect and reduce the complications [27]. Feng et al. reported that MC showed better effect on new bone regeneration in alveolar ridge preservation [28]. Based the recognition of MC, we assessed the first application of MC in IIP using retrospective data. Herein, the purpose of this study was to evaluate the therapeutic effect of MC by radiological analysis. We hypothesized that application MC would achieve a satisfactory clinical outcomes in IIP.

## Methods

### Study design; in-/exclusion criteria

Medical records were selected among those patients who had been treated with immediate post-extraction implants and performed GBR protocol using bone grafting either MC (Allgens®, Beijing Allgens Medical Science and Technology Co., Ltd., China) or Bio-Oss (Bio-Oss®, Geistlich Biomaterials, Wolhusen, Switzerland) at the Department of oral and maxillofacial surgery of Shandong Provincial Hospital Affiliated to Shandong University (Jinan, Shandong Province, China), in the period between January 2018 and December 2019.

### Inclusion criteria

1. Aged ≥ 18.
2. Class I and class II B of Gluckman’s classification [29].
3. No acute infection at the extraction site [30].
4. Sufficient volume of apical and palatal bone at the extraction site to allow implant placement in a correct 3D position with primary stability [30].
5. Good general health.
6. Good oral hygiene.

Especially, patients included in this study had to be followed for a minimum period of 1 year after surgery.

### Exclusion criteria

1. Contraindication for surgery such as uncontrolled diabetes, pregnancy, previous or current radiation or immunosuppressive therapy.
2. Smoking (over ten cigarettes per day) and excessive drinking.
3. Patients with any systemic disease that could affect bone healing were excluded from the study.

Patients were divided into two groups: the MC group who were grafted with MC and the Bio-Oss group with Bio-Oss. All the treatments were arranged consecutively at the Department of Oral maxillofacial surgery of Shandong Provincial Hospital Affiliated to Shandong University. Patients were informed about the treatment purpose, process, and the possible risks of this study, and then signed the informed consent.

### Surgical procedure

In order to evaluate the extraction sites, clinical examination and CBCT were taken for each patient. Periodontal treatment and oral hygiene instructions were treated to all patients for better oral environment. Each patient was received antibiotics (Roxithromycin Capsules of 150 mg) 1 h before surgery and rinsed with 0.2% chlorhexidine gluconate for 1 min.

All patients were performed by Dr. Huang. Articaine (4% articaine with 1:100,000 epinephrine) was used as local anesthetic. A flap was designed with the implant site followed by sulcular incisions on the buccal and palatal that extends one tooth to the mesial and distal without vertical incisions. The flap didn’t exceed the mucogingival junction. The tooth was extracted using atraumatic extraction technique and the granulation tissue was carefully removed with curette. Following the instructions of implant manufacturer, the preparation of the implant site was performed rinsed with abundant sterile saline. The pilot drill was conducted to the palatal wall. According to socket size, implant diameter was determined to achieve > 1.5 mm mesio-distal implant distance and the length of implant was 11–13 mm. The implant was inserted with 35 Ncm or more and 1 mm below the most apical bone peak. Then the healing abutment was placed. In all cases (not related to the socket configuration or defect morphology), bone substitute material was applied in the residual gap. In MC group, surgery was performed using MC, while Bio-Oss group was placed with Bio-Oss. CBCT (ProMax 3D, Planmeca OY, 00880 Helsinki, Finland) scans for each patient were performed to confirm proper implant placement after surgery.

Patients were postoperatively instructed to take roxithromycin (150 mg × 2/day for 5–7 days) and 0.2% chlorhexidine gluconate twice a day for 14 days.

### Restorative procedure

The reconstructive treatment protocol was performed 6 months after implantation. The patients were scheduled to take CBCT to observe the alveolar bone change. Standard implant impression was made using silicon rubber impression material (DMG, Hamburg, Germany) for permanent restorations. All clinical procedures of permanent restoration were operated by Dr. Han.

### Clinical follow-up

The time immediately postsurgery, 6 months and 12 months after permanent restoration were respectively set as T1, T2, and T3. Patients were recalled at T3 to record implant survival rate and give a clinical examination.

### Evaluation criteria and methods

#### Implant survival and complications

Implant survival and complications were observed at 12 months after the final crown delivery. According to Buser's criteria for successful implant osseointegration [31], the success criteria are as follows:

- No persistent subjective complaints, such as pain, and/or paresthesia
- No recurrent peri-implant infection with suppuration
- No mobility
- No continuous radiolucency around the implant
- Possibility for restoration

#### Radiographic measurements

CBCT was scheduled to assess the osseointegration process at T1, T2, and T3 followed by Yang et al. [32]. Specific fixed reference points were selected in the images to obtain the same tri-dimensional position of the measurement axis. The reference landmarks were defined as follows.

- Implant shoulder (I).
- Top of the bone crest (C).
- Implant shoulder to labial bone crest (OC).
- Horizontal distance between OC and I (OCI).
- Vertical distance between I and C (ICH).

**Fig. 1:**
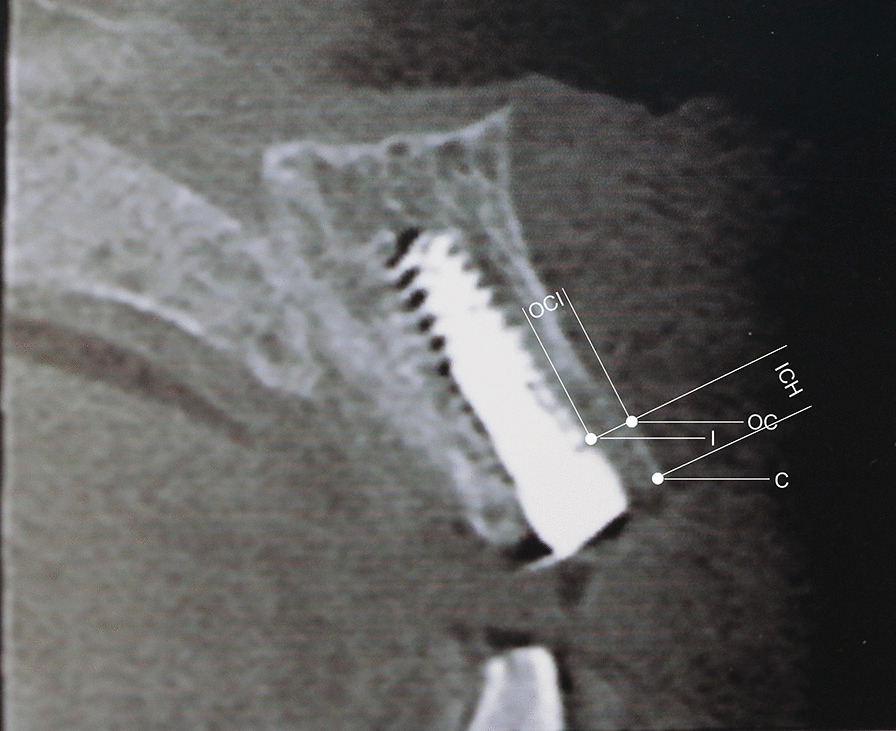
Illustration of radiographic measurement

Dimension changes of labial bone were measured by OCI and ICH at T2and T3 compared to T1. This measure defined as ΔOCI and ΔICH was taken at least three times, and the mean values were recorded (Fig. 1).

### Esthetic assessment

All 70 patients were scheduled to recall at T3. A digital camera (Canon 6D, Canon Inc., Tokyo, Japan) was used with the same parameters to obtain standardized digital photographic records which were taken at the occlusal plane and centered at the contact region of the upper incisors.

The esthetic outcome and performance were evaluate by the pink esthetic score (PES) described by Belser et al. [33]. The PES contains the following five variables: mesial papilla, distal papilla, curvature of the facial mucosa, level of the facial mucosa, root convexity/soft tissue color and texture at the facial aspect of the implant site. A score of 2, 1, or 0 is assigned to the parameters above. Thus, the maximum score for optimal soft tissue is 10. The PES was objectively determined by three independent examiners who were not involved in the surgical procedure.

### Visual analog scale (VAS) evaluation of patient satisfaction

Patient satisfaction related to the esthetic outcome, pain, and swelling was measured with VAS by filling out a questionnaire at T3. Answers were recorded ranging from 0 to 10 labeled with “0 = totally unsatisfied, no pain, no swelling” and “10 = fully satisfied, extreme pain, extreme swelling” [34].

### Statistical analysis

Data analysis was performed using SPSS 16.0 software (SPSS Inc., Chicago, IL, USA). Results were demonstrated as the mean ± standard deviation (SD). The t-test was used to compare the variables. *P* < 0.05 was considered statistically significant.

## Results

Overall, 70 patients (34 women, 36 men) with a mean age of 40.1 ± 12.3 were included in the present retrospective study and they received a total number of 80 implants. There were no statistically significant differences in patient age, gender, smoking history, implant brand and implant site in two groups (Tables 1, 2). According to Buser's criteria for successful osseointegration, all 80 implants remained well-integrated with a 100% implant survival rate after 1 year of permanent restoration. Furthermore, no significant differences were seen in bone graft types within the two groups.

**Table 1:** Demographic information of the MC group

Patient no	Implant brand	Implant site	Implant dimension (mm)	Insertion torque (Ncm)	healing abutment/temporary crown
1	XIVE®	11 21	3.8 × 13 3.8 × 13	35	Temporary crown
2	NobelActive®	11	4.3 × 13	35	Temporary crown
3	XIVE®	11	3.8 × 13	30	Healing abutment
4	XIVE®	21	3.8 × 13	35	Temporary crown
5	XIVE®	11	3.8 × 13	30	Healing abutment
6	NobelActive®	22	3.5 × 11.5	35	Temporary crown
7	XIVE®	11	3.8 × 13	40	Temporary crown
8	NobelActive®	21	3.8 × 11	35	Temporary crown
9	XIVE®	11	3.8 × 13	35	Temporary crown
10	NobelActive®	21 22 23	4.3 × 11.5 4.3 × 11.5	35	Temporary crown
11	XIVE®	21	3.8 × 13	35	Temporary crown
12	NobelActive®	21	4.3 × 13	25	Healing abutment
13	NobelActive®	11 21	4.3 × 11.5 4.3 × 11.5	35	Temporary crown
14	XIVE®	12	3.4 × 11	35	Temporary crown
15	NobelActive®	12	3.4 × 11.5	35	Temporary crown
16	XIVE®	11 21	3.8 × 13 3.8 × 13	35	Temporary crown
17	XIVE®	11	3.8 × 13	30	Healing abutment
18	XIVE®	21	3.8 × 13	40	Temporary crown
19	NobelActive®	21	4.3 × 11.5	35	Temporary crown
20	NobelActive®	11	3.5 × 13	30	Healing abutment
21	XIVE®	12	3.8 × 11	35	Temporary crown
22	XIVE®	11	3.8 × 13	25	Healing abutment
23	XIVE®	12	3.4 × 13	30	Temporary crown
24	XIVE®	21 22	3.8 × 13 3.4 × 13	30	Temporary crown
25	NobelActive®	13	4.3 × 13	35	Healing abutment
26	XIVE®	11	3.8 × 13	35	Temporary crown
27	NobelActive®	21	43 × 13	30	Healing abutment
28	NobelActive®	22	3.5 × 11.5	30	Healing abutment
29	XIVE®	12	3.4 × 11	35	Healing abutment
30	XIVE®	21	3.8 × 13	35	Temporary crown
31	XIVE®	12	3.8 × 11	35	Temporary crown
32	XIVE®	11	3.8 × 13	25	Healing abutment
33	XIVE®	21	3.4 × 13	30	Healing abutment
34	XIVE®	11	3.8 × 13	35	Temporary crown
35	XIVE®	11	3.8 × 13	35	Temporary crown

**Table 2:** Demographic information of the Bio-oss group

Patient no	Implant brand	Implant site	Implant dimension (mm)	Insertion torque (Ncm)	Healing abutment/temporary crown
1	NobelActive®	11	4.3 × 11.5	35	Temporary crown
2	NobelActive®	21	4.3 × 13	35	Temporary crown
3	XIVE®	21	3.8 × 13	35	Temporary crown
4	XIVE®	11 21	3.8 × 13 3.8 × 13	35	Temporary crown
5	NobelActive®	22	3.5 × 11.5	35	Temporary crown
6	XIVE®	11	3.8 × 13	30	Healing abutment
7	XIVE®	11	3.8 × 13	35	Temporary crown
8	XIVE®	11 21	3.8 × 13 3.8 × 13	30	Healing abutment
9	XIVE®	21	3.8 × 13	35	Temporary crown
10	XIVE®	12	3.8 × 13	35	Temporary crown
11	NobelActive®	21 22	4.3 × 11.5 4.3 × 11.5	35	Temporary crown
12	XIVE®	11	3.8 × 13	40	Temporary crown
13	XIVE®	21	3.8 × 13	40	Temporary crown
14	NobelActive®	21	4.3 × 11.5	35	Temporary crown
15	XIVE®	12	3.8 × 11	35	Temporary crown
16	XIVE®	11	3.8 × 13	25	Healing abutment
17	NobelActive®	11	4.3 × 13	35	Temporary crown
18	XIVE®	21	3.8 × 13	35	Temporary crown
19	XIVE®	11 21	3.8 × 13 3.8 × 13	35	Temporary crown
20	XIVE®	11	3.8 × 13	30	Healing abutment
21	XIVE®	11	3.8 × 13	25	Healing abutment
22	XIVE®	22	3.4 × 13	30	Healing abutment
23	NobelActive®	13	4.3 × 13	35	Temporary crown
24	NobelActive®	12	3.5 × 11.5	30	Healing abutment
25	XIVE®	22	3.4 × 11	35	Healing abutment
26	NobelActive®	21	3.8 × 11	35	Temporary crown
27	NobelActive®	21	43 × 13	30	Healing abutment
28	XIVE®	21	3.8 × 13	35	Temporary crown
29	XIVE®	12	3.8 × 11	35	Temporary crown
30	XIVE®	11	3.8 × 13	25	Healing abutment
31	NobelActive®	12	3.5 × 11.5	35	Temporary crown
32	XIVE®	11 13	3.8 × 13 3.8 × 13	35	Healing abutment
33	XIVE®	11	3.8 × 11	35	Temporary crown
34	NobelActive®	21	3.5 × 13	35	Temporary crown
35	XIVE®	21	3.8 × 11	35	Temporary crown

### Radiographic outcomes

The alveolar bone in both groups showed insignificant differences in horizontal and vertical bone loss at T2 and T3. As the specific data demonstrated in Table 3, the differences of OCI and ICH between the MC group (0.72 ± 0.26 mm, 1.62 ± 0.84 mm) and the Bio-Oss group (0.70 ± 0.52 mm, 1.57 ± 0.88 mm) were statistically no significant at T2. Similar results occurred at T3. The reduction of OCI and ICH were 0.68 ± 0.91 mm and 1.55 ± 1.05 mm in MC group, and the Bio-Oss group showed 0.62 ± 0.78 mm and 1.49 ± 0.90 mm loss in OCI and ICH.

**Table 3:** Dimensional changes at T2 and T3 by CBCT measurements

Variable	6 months		12 months	
	ΔOCI	ΔICH	ΔOCI	ΔICH
	Mean (mm)		Mean (mm)	
MC group	0.72 ± 0.26	1.62 ± 0.84	0.68 ± 0.91	1.55 ± 1.05
Bio-Oss group	0.70 ± 0.52	1.57 ± 0.88	0.62 ± 0.78	1.49 ± 0.90
*P* value	0.90	0.88	0.87	0.66

OCI indicated the thickness of the labial crest (including bone graft) at the level of implant shoulder

ICH indicated the relative height of the labial crest at the level of implant shoulder

In addition, compared T2 to T3, the resorption of alveolar bone at horizontal distance (OCI) and vertical distance (ICH) in MC group or in Bio-Oss group had no statistically significant.

### Esthetic outcomes

Overall, none of the five parameters of the PES, or the total PES (6.07 ± 1.62 vs. 6.13 ± 1.41) values were significantly different when comparing the MC and the Bio-Oss group 1 year after crown placement (Table 4). What’s more, the percentage of clinical acceptance was 60% in both groups.

**Table 4:** Comparison of PES values at T3 after crown placement

Group	Mesial papilla	Distal papilla	Curvature of facial mucosa	Level of facial mucosa	Root convexity/soft tissue color and texture	Total score (maximum10)	Acceptable (100%)
	Mean ± SD	Mean ± SD	Mean ± SD	Mean ± SD	Mean ± SD	Mean ± SD	
MC group	1.13 ± 0.74	1.27 ± 0.59	0.87 ± 0.52	1.40 ± 0.74	1.40 ± 0.63	6.07 ± 1.62	60%
Bio-Oss group	1.2 ± 0.77	1.20 ± 0.68	0.93 ± 0.70	1.33 ± 0.72	1.47 ± 0.64	6.13 ± 1.41	60%
*p* value significance	0.81, ns	0.78, ns	0.77, ns	0.80, ns	0.81, ns	0.91, ns	

Clinical acceptability was defined as PES ≥ 6

*ns* non-significant

### VAS outcomes

Patient satisfaction estimated by VAS was similar (8.56 ± 1.12 vs. 8.27 ± 1.44), and the difference was not statistically significant (*P* = 0.538; Table 5) between the two groups.

**Table 5:** The outcomes of patient satisfaction

	MC group	Bio-Oss group	*p* value
	Mean ± SD	Mean ± SD	
VAS score	8.56 ± 1.12	8.27 ± 1.44	0.538

## Discussion

In this retrospective study, our hypothesis was that application of MC in IIP could achieve a similar effect as Bio-Oss. The results revealed that no significant differences were found in terms of clinical and radiographic assessments, as well as esthetic outcomes and patient satisfaction at 12 months after permanent restoration. Therefore, our hypothesis for this study was accepted.

It is well known that buccal bone was concerned to be one of the most important features when it comes to get satisfying aesthetic results. It has also been claimed that the “critical thickness” value to the buccal bone should be at least 2 mm thick [35]. In addition, bone grafting materials in combination with IIP achieved better osseointegration in comparison to a situation where IIP was done without bone grafting [36]. Therefore, the chosen grafting materials to solve bone defect for new bone formation should be concerned at present.

Bio-Oss® deproteinized bovine bone with excellent characteristic of osteoconductive property, high biocompatibility, and low biodegradation rate has been described as the most successful bone substitute worldwide and remains the best choice in a variety of graft materials.

Biomimetic artificial MC designed by Cui and his co-workers is an artificial bone graft which mimics composition and microstructure of human natural bone [26, 37]. Its remarkable treatment effect on bone defect reparation has been confirmed in more than 200,000 cases in clinical area of orthopedics, stomatology, neurosurgery and so on [27]. Many in vitro animal studies also have been verified the biocompatibility, biodegradability and osteoconductive potency of MC [38, 39]. For example, Ghate et al. demonstrated that MC applied for the patient with collapse and subluxation of metatarsal-cuneiform joint acted as an excellent alternative to autograft in fusing the podarthral joints with internal fixation [40]. Liu et al. also investigated the effect of MC on the rabbit rib defect, and reported that the MC group showed a higher bone remodeling activity in comparison with blank control group [41]. Moreover, Wu et al. proved that osteogenic differentiation on MC incorporated in poly bone cement was more than two times higher than that of poly alone after culturing for 21 day in human marrow mesenchymal stem cells culture system [42]. The result confirmed the important mechanism on osteogenic properties of MC at cellular level.

Preferably, bone grafts should be gradually degraded and replaced with newly-formed bone. As previous literature has reported, MC bone grafts should be a promising alternative for bone augmentation in oral surgeries. To the best of our knowledge, there are no available studies to confirm the effect of MC in promoting bone regeneration in the treatment of IIP. Therefore, in the present study, we firstly used the biomimetic MC as bone grafting material. As results shown above, 70 patients had been treated with 80 implants, and the implant survival rate was 100% in both groups. Additionally, the radiographic analysis revealed that no significant difference in bone reduction between the two groups at T2 and T3. Furthermore, the results of esthetic and patient satisfactory were also consistent with radiographic findings. Similarly, Wang et al. compared the clinical and histologic effect of Bio-Oss and MC bone materials on minipig, and found that the MC achieved the similar result as Bio-Oss [43]. Although there is no investigation comparing the efficacy of MC with that of Bio-Oss in IIP, our findings were similar to the results of studies above.

Nevertheless, the present study has some limitations, including small sample size (n = 70), short follow-up period, and lack of bone biopsy for histological evidence. Within its limits, further studies with larger sample sizes and long-term investigation are required for corroboration of these findings.

## Conclusions

In conclusion, this study demonstrated that the biomimetic MC showed a similar behaviour as Bio-Oss not only in its dimensional tissues changes but also in clinical acceptability and patient satisfaction. Within the limitations of this study, these cases show that MC could be considered as an alternative bone graft in IIP.

## Case report

**Fig. 2:**
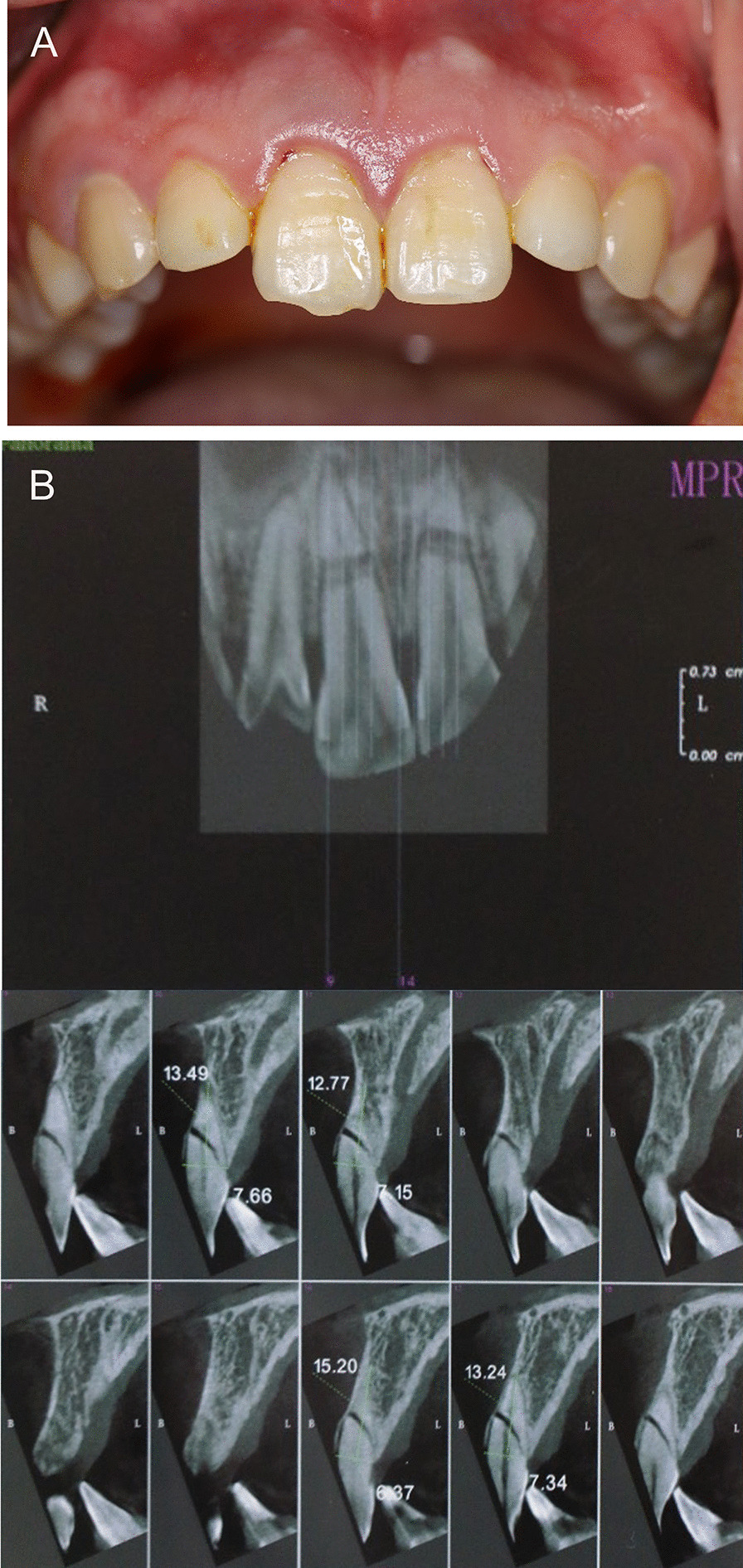
Preoperative view. **A** Intraoral photograph, **B** preoperative CBCT

A 28-year-old healthy female with root-fractured anterior teeth was referred to our hospital. The teeth (#11, #21) showed 2° mobility and had slight tenderness on percussion. Radiographic examinations revealed that #11 and #21 were root-fractured at the apical third (Fig. 2).

**Fig. 3:**
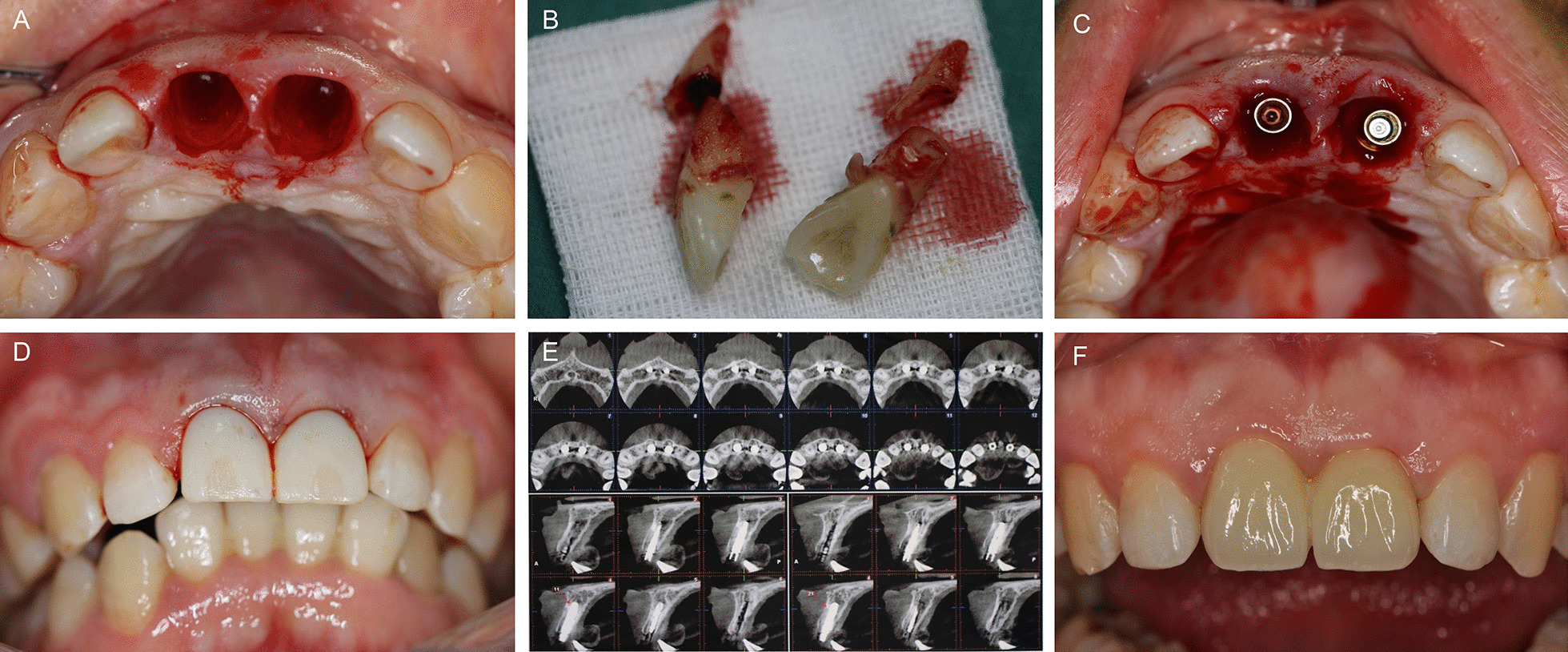
Demonstration of the details of the process. **A** Atraumatic extraction of 11, 22, **B** extracted crown and root fragments, **C** immediate implant placement, **D** the temporary crown immediately after operation, **E** postoperative CBCT, **F** permanent restoration

Considering the region of high esthetic value and patient’s bone quantity, we decided to proceed with implanting #11, #21 immediately after teeth extraction, using XIVE® implant system (XIVE®, Dentsply Friadent, Mannheim, Germany) replacement and MC for bone augmentation. Afterwards, the patient was treated by immediate reconstruction and permanent restoration was fixed 4 months postoperatively (Fig. 3A–F).

## Data Availability

All the datasets and materials used and analyzed during the current study are included in this published article. The datasets used and analyzed during the current study are available from the corresponding author on reasonable request.
